# Telephone-triage services do not lead to an increased wait time for assessment of gonorrhoea in symptomatic patients

**DOI:** 10.1177/0956462421999280

**Published:** 2021-05-05

**Authors:** William Jasper, Madeleine Macdonald, Danayan Luxmanan, Elizabeth Foley, Rajul Patel

**Affiliations:** 1 Genitourinary Medicine, Faculty of Medicine, 7423 University of Southampton, Southampton, UK; 2 Solent Sexual Health, 232267 Royal South Hants Hospital, Southampton UK

**Keywords:** Gonorrhea (*Neisseria gonorrhoeae*), bacterial disease, urethritis (non-specific), other, high-risk behaviour

## Abstract

In Spring 2017, Southampton and Portsmouth Sexual Health Services (SHSs) replaced an overstretched walk-in service with a telephone-triage service: patients calling that were symptomatic, vulnerable or at high risk of having an STI were invited into a clinic, whereas others were signposted to remote self-sample NHS postal testing services. This study aimed to establish whether patient care was disadvantaged by the introduction of the triage service. Electronic patient notes for all patients attending for treatment of gonorrhoea for two years before and for two years after the service change were interrogated; the site of infection and duration of symptoms before testing were compared. Of all patients attending for treatment of gonorrhoea in the study period, 499 patients (39% of cases) were symptomatic at testing: 364 had urethral symptoms, 45 had rectal symptoms and 18 had pharyngeal symptoms. 72.4% of patients with urethral symptoms were seen after the introduction of the triage system. Median wait times for patients with urethral symptoms rose from 6 (IQR = 3–7) to 7 (IQR = 3.75–14) days – although this increase was not statistically significant (*p* = 0.064). There was not a statistically significant difference between the rectal symptom groups (*p* = 0.422) and too few patients attended with pharyngeal symptoms to warrant analysis. Despite some outliers, the telephone-triage service did not increase wait times for patients attending STI services with symptomatic gonorrhoea and may have inadvertently increased access to services for those most at risk.

## Background

Sexual health services (SHSs) are under ever-increasing pressure, evidenced by rising patient numbers^1^ and reductions in public health budgets.^2^ Many services have looked to innovate to improve efficiency; there has been investment in remote sample collection, attempts to replace open-access services with telephone-based triage and sign posting face-to-face services to those most likely to benefit from them. In 2017, Southampton and Portsmouth SHSs (Royal South Hants Hospital and St Marys Community Health Campus) adopted a telephone-triage service. This replaced the previous open-access walk-in service for STI testing, where patients were able to attend the service and expect consultation with testing. The walk-in clinics created a chaotic work environment for staff and long waits for patients – often up to several hours long. The new system replaced walk-in appointments, and instead, patients were triaged over the phone. Those that were symptomatic, vulnerable or at high risk of having an STI were invited into the clinic at a fixed time for testing. Those that did not fit these criteria were sent free self-sampling tests to perform home-based testing and were not invited into the clinic unless test results were positive.

## Aim

The aim of this study was to establish whether patient care was disadvantaged by the introduction of the telephone-triage service.

## Methods

To measure the impact of the new telephone-triage service, the duration of symptoms in patients before testing was compared before and after the introduction of the telephone-triage service. Symptomatic gonorrhoea infection was chosen as the indicator illness as most patients with symptomatic gonorrhoea infection present within 2 to 5 days of infection.^3^

Electronic patient notes for all patients treated for gonorrhoea infection in Portsmouth (Centre 1) and Southampton (Centre 2) were accessed. All patients treated within the 2 years before and for the 2 years after the introduction of the telephone-triage service were included in the study. Data were collected covering basic demographics, duration and nature of symptoms, centre of testing and method of presentation to the service (walk-in or telephone triage). Symptoms were categorised based on the description of gonorrhoea infection in the BASHH guidelines.^3^ The study was approved by the University of Southampton Ethics and Research Committee (ERGO number: 49,673).

## Results

All unique cases of gonorrhoea attending over the 4 year study period in Solent were reviewed. 499/1279 patients attending for treatment of gonorrhoea (39% of all cases) were symptomatic at presentation. 364 (72.9%) of these had urethral symptoms, 45 (9%) had rectal symptoms and 18 (3.6%) had pharyngeal symptoms. Table 1 shows the distribution of symptoms for the patients that attended the service with symptomatic gonorrhoea infection throughout the study period.

**Table 1.** Frequency of symptoms as experienced by patients with symptomatic gonorrhoea throughout the study period.

Symptom	Frequency
Urethral (discharge and/or dysuria)	364 (72.9%)
Rectal	45 (9%)
Pharyngeal	18 (3.6%)
Menstrual changes	7 (1.4%)
Testicular/epididymal tenderness	5 (1%)
Superficial vaginal tenderness/pain	5 (1%)
Superficial penile redness/irritation/pain	8 (1.6%)
Not a common symptom of gonorrhoea	26 (5.2%)
Too vague to state	8 (1.6%)
Abdominal pain	10 (2%)
Dyspareunia/post-coital bleeding	1 (0.2%)
Ophthalmia	1 (0.2%)
Change in urination frequency	1 (0.2%)

Across the three commonest symptoms, 54 patients had been diagnosed through alternative services prior to their attendance in the clinic. Of the patients diagnosed after attendance in service (445/499; 89.2%), only those patients where a clear duration of symptoms could be calculated from the notes were further analysed. Table 2 contains the demographics of patients presenting with urethral and rectal symptoms that attended for STI testing that had their duration of symptoms recorded. As only one patient with pharyngeal symptoms presented to the walk-in service and had their duration of symptoms recorded, pharyngeal symptom data have not been included as comparison was not deemed appropriate.

**Table 2.** Centre attended and demographics of patients with urethral and rectal symptoms where duration of symptoms were recorded.

Demographic	Urethral symptom		Rectal symptom	
	Walk-in service (^a^)	Triage service (^a^)	Walk-in service (^a^)	Triage service (^a^)
Sex				
Male	51 (70.8%)	137 (75.3%)	7 (77.8%)	14 (50%)
Female	9 (52.9%)	30 (57.7%)	0	0
Sexual orientation				
Heterosexual	23 (60.5%)	119 (72.6%)	0	0
Gay	28 (70%)	39 (66.1%)	7 (77.8%)	12 (52.2%)
Lesbian	0	0	0	0
Bisexual	7 (87.5%)	6 (75%)	0	1 (25%)
Missing	2 (66%)	3 (100%)	0	1 (100%)
Mean age [years old]	31.2 [SD = 11.8]	29.08 [SD = 10.7]	33.1 [SD = 11.7]	30.6 [SD = 8.24]
Ethnicity				
White British	40 (64.5%)	99 (67.8%)	6 (100%)	10 (50%)
Mixed	1 (50%)	6 (54.5%)	0	0
Black/Black British	5 (71.4%)	26 (89.7%)	0	0
Asian/Asian British	6 (100%)	9 (75%)	0	1 (100%)
White other	3 (60%)	9 (69.2%)	1 (50%)	2 (66.7%)
Other	0	1 (100%)	0	0
Missing	5 (71.4%)	17 (77.3%)	0	1 (25%)
Centre attended				
Centre 1	23 (60.5%)	98 (73.7%)	1 (50%)	7 (63.6%)
Centre 2	37 (72.5%)	69 (68.3%)	6 (85.7%)	7 (41.2%)

^a^The proportion of patients of each category that had their duration of symptoms recorded.

In the urethral symptom group (patients with urethral symptoms that had their duration of symptoms recorded), 60 patients attended the walk-in service and 167 attended via the telephone-triage service. Between the two groups, the median time from symptoms to testing increased from 6 to 7 days from the walk-in group to the triage group; this was not statistically significant (Mann–Whitney *U* test, *p* = 0.064). The mode for both groups was 7 days and the interquartile range increased from 3–7 days to 3–14 days.

In the rectal symptom group (patients with rectal symptoms that had their duration of symptoms recorded), seven patients attended the walk-in service and 14 attended via the telephone-triage service. The median wait appeared to increase from 7 to 10 days; however, this was also not statistically significant (Mann–Whitney *U* test, *p* = 0.422). The mode increased from 7 to 14 days and the interquartile range remained relatively unchanged (5–14 days to 6.5–14 days).

As stated, two patients attended the walk-in service with pharyngeal gonorrhoea symptoms; however, only one had their duration of symptoms noted. 11 patients attended the triage service with pharyngeal symptoms, and six of these had their duration of symptoms recorded. Due to such low numbers attending the walk-in service, comparison was not deemed appropriate.

## Discussion

The lack of a statistically significant difference between the walk-in and telephone-triage services for patients with urethral and rectal symptoms indicates that patients are not disadvantaged by the introduction of the telephone-triage service. The triage service may have inadvertently increased the accessibility of STI services for those with common symptoms of gonorrhoea infection. The number of patients attending through the triage service with urethral symptoms was 160% greater than the number of patients attending the walk-in service. This may reflect an increase in STI diagnoses nationally. However, the rate of increased diagnosis in Solent exceeded the national rise of gonorrhoea diagnoses between 2014 and 2018 (an increase of 51.3%)^4^ and may be an indicator of the inability of the system to cope with demand in 2017 as patients may have been put off by long waiting times in the walk-in service. This perhaps suggests that symptomatic patients were more willing to attend services in the knowledge that they will not be spending many hours waiting in the clinic.

There was a minority of patients who waited longer in the triage group than the walk-in group. This is seen by the increased maximum wait times (for both the urethral and rectal symptom groups) and increased interquartile range (urethral symptom group). This could reflect an unwillingness or inability of patients to vocalise symptoms over the phone or even indicate that patients find using the telephone a barrier to access.

There were some limitations to this study. The greatest of which was the quality of documentation in the patient notes. There were several cases where there was no mention of symptom status or the duration of symptoms (up to 32.6% of patients attending with urethral symptoms in the walk-in group did not have their duration of symptoms recorded). This impacted the quality of research, but also fails to provide adequate records of patient assessment. Furthermore, there was no measure of symptom severity. Less severe symptoms may have led some patients in the triage groups to wait longer before seeking advice especially if they considered them trivial – these may be the patients that the walk-in system failed to reach.

Women made up only one-fifth of the study population and rarely present with the symptoms we have used for markers of service delivery. The results cannot be extended to them or to any of the rarer symptomatic presentations of genital infection.

## Conclusion

As more patients require SHSs than ever before, there is a growing need to provide more efficient services without disadvantaging patient care. Telephone-triage services provide a cost-effective strategy for limiting face-to-face consultations compared with an open access walk-in clinic. This study has established that telephone triage does not significantly increase the wait times for patients with common symptoms of gonorrhoea attending for STI testing and may increase accessibility of STI services.
